# Hepatoprotective Effect of Curcumin Nano-Lipid Carrier against Cypermethrin Toxicity by Countering the Oxidative, Inflammatory, and Apoptotic Changes in Wistar Rats

**DOI:** 10.3390/molecules28020881

**Published:** 2023-01-16

**Authors:** Sohail Hussain, Mohammad Ashafaq, Saeed Alshahrani, Ibrahim A. M. Bokar, Rahimullah Siddiqui, Mohammad Intakhab Alam, Manal Mohamed Elhassan Taha, Yosif Almoshari, Saad S. Alqahtani, Rayan A. Ahmed, Abdulmajeed M. Jali, Marwa Qadri

**Affiliations:** 1 Department of Pharmacology and Toxicology, College of Pharmacy, Jazan University, Jazan 82817, Saudi Arabia; 2 Pharmaceutical Science in Applied Toxicology, College of Pharmacy, Jazan University, Jazan 82817, Saudi Arabia; 3 Department of Pharmaceutics, College of Pharmacy, Jazan University, Jazan 82817, Saudi Arabia; 4 Substance Abuse Research Center (SARC), College of Pharmacy, Jazan University, Jazan 82817, Saudi Arabia; 5 Clinical Pharmacy Department, College of Pharmacy, Jazan University, Jazan 82817, Saudi Arabia; 6 Pharmacy Practice Research Unit, College of Pharmacy, Jazan University, Jazan 82817, Saudi Arabia

**Keywords:** curcumin, cypermethrin, oxidative stress, inflammatory cytokines, hepatotoxicity

## Abstract

This study investigated the potential hepatoprotective activity of curcumin-incorporated nano-lipid carrier (Cur-NLC) against cypermethrin (Cyp) toxicity in adult Wistar male rats. All animals in groups III, IV, V, and VI were subjected to Cyp (50 mg/kg) toxicity for 15 days. Three different doses of Cur-NLC (1, 2.5, and 5 mg/kg/day) were administered orally for 10 days. The toxic effects were evaluated considering the increases in serum hepatic biomarkers alanine aminotransferase (ALT), aspartate aminotransferase (AST), alkaline phosphatase (ALP), total protein and albumin, and lipid peroxidation (LPO), as well as a decrease in antioxidative activity (reduced glutathione (GSH), superoxide dismutase (SOD), and catalase) and the upregulation of inflammatory cytokines (IL-1β, IL-6, and TNF-α). Immunohistochemistry studies of proteins (NF-κB, Apaf-1, 4-HNE, and Bax) showed enhanced expression, and histopathological examination revealed architectural changes in liver cells, indicating liver toxicity in animals. Toxicity was determined by quantitative and qualitative determinations of DNA fragmentation, which show massive apoptosis with Cyp treatment. The administration of Cur-NLC significantly ameliorates all changes caused by Cyp, such as a decrease in the levels of serum liver markers, an increase in antioxidative parameters, a decrease in expression of inflammatory cytokines (IL-1β, IL-6, TNF-α, and NF-κB), and apoptosis (caspases-3, 9, Apaf-1, 4-HNE, and Bax), according to calorimetric and immunohistochemistry studies. The smear-like pattern of DNA is ameliorated similarly to the control at a high dose of Cur-NLC. Furthermore, all histopathological changes were reduced to a level close to the control. In conclusion, Cur-NLC could be a potent nutraceutical that exhibits a hepatoprotective effect against Cyp-induced hepatotoxicity in rats.

## 1. Introduction

Currently, pesticide toxicity is a major global health problem [1] and causes deleterious health-related issues in humans and animals. The exposure of humans and animals to pesticides occurs mainly through environmental contamination in dairy products, animal feed, and water.

Most Cyp is absorbed into our body through the digestive tract. Cyp is present in cis and trans isomeric configurations and produces phenoxybenzoic acid and cyclopropanecarboxylic acid upon metabolism [2]. In humans, Cyp is eliminated through urine and will last up to 48 h, but previous work reported that hydroxylation and cleavage are the main metabolic steps, with over 99% excreted within 60 min. Approximately 1% is stored in body fat and slowly eliminated. About 18 days and 3–4 days are the half-life of cis and trans isomers, respectively [3]. However, Cyp remains for almost three months in the air, on walls, and on furniture [4]. However, there are some reports of mammalian toxicity resulting from accumulation in various tissues [5,6]. Cyp can pass through the cell membrane because of its hydrophobic property. It disturbs the membrane structure and causes leakage of cytoplasmic enzymes into the bloodstream [6].

Cyp is metabolized by the enzyme cytochrome P450, which involves oxidation, hydrolysis, conjugation reactions, and ester cleavage in the liver [7]. The major mechanism of action of Cyp is extremely complex and mostly acts through modulating the sodium–potassium channels by delaying its action [8]. Although the key target of Cyp is neuronal cells, several studies have reported that it may be linked to hepatotoxicity and genotoxicity [7,9]. Cyp is mainly metabolized by liver enzymes; hence, the liver is considered as a secondary target organ. Cyp metabolites may lead to hepatic cell damage, producing reactive oxygen species (ROS); thus, oxidative stress is caused [10]. Furthermore, oxidative stress leads to oxidative damage of lipid content, protein oxidation, and DNA damage [11], which further activate inflammation and apoptosis in the liver [12]. 

The toxic properties of Cyp are mostly mediated through the production of ROS [13]. This causes an imbalance in oxidative stress by increasing LPO and decreasing enzymatic activities (SOD, catalase) and non-enzymatic GSH [14]. ROS react with cellular components and macromolecules and make them unstable. A recent study established that ROS further activate inflammation and apoptosis through NF-κB, IL-1β, IL-6, caspase 3, 9, Bax, 4-HNE, and apaf-1 [15]. Inflammatory markers (NF-κB, IL-1β, and IL-6) also play an important role in Cyp toxicity [16]. Inflammation is a complex process elicited by ROS and reported in the initiation and progression of liver diseases [17,18]. NF-κB is a primary transcription factor enhanced due to ROS during liver damage, which induces activation of various other proinflammatory genes that include IL-1β and IL-6 [19]. Apoptosis can also be evaluated using DNA fragmentation by Cyp [20]. Extensive DNA breakdown by Cyp in the brain was reported by DNA laddering [21]. Recently, researchers have been working on natural products or medicinal plants for the treatment of free radical toxicity because they are less expensive and have no harmful side effects as compared to modern medicine [22]. Molecules with antioxidative activity react with ROS to neutralize them, reduce oxidative stress, and finally protect against diseases [23].

Curcumin is a yellow pigment, lipophilic in nature, that is extracted from the roots of turmeric plants (*Curcuma longa* of the ginger family (Zingiberaceae)) and mainly used in food coloring and the food industry, and was used in ancient medicine [24,25]. Previous research by Elsayed et al. (2016) proved that curcumin is beneficial as an anti-carcinogenic, anti-tumoral, anti-inflammatory, and hepatoprotective natural substance [26]. Curcumin compounds were proposed to have a protective effect on Cyp-induced liver toxicity [27]. Curcumin is a potent scavenger of ROS including superoxide anion radicals and hydroxyl radicals [28]. Curcumin administration is also effective in various animal models, where it prevents oxidative stress and the development of free radicals as a result of organ toxicity [29,30]. The earlier studies confirm that curcumin nano formulation enhanced their activities in different animal models of the liver and kidneys [31,32]. Curcumin alone exhibits poor bioavailability due to its hydrophobic activity. In agreement with the earlier studies, our group developed a curcumin nano formulation which has a relatively excellent distribution in the liver. Thus, the formulation of a curcumin-incorporated nano-lipid carrier (Cur-NLC) could be an advanced strategy to increase its bioavailability, which is why it reduces the required dose against hepatotoxicity. 

Therefore, in this work, we hypothesized that Cur-NLC prevents hepatotoxicity induced by Cyp through ameliorating oxidative stress, inflammation apoptosis, and histological alteration. 

## 2. Results

### 2.1. Serum Biochemical Assay

A marked increase (*p* < 0.001) in the activities of ALT, AST, and ALP enzymes was found in group III, which was treated with Cyp alone, as compared with the control (group I), while co-treatment with varying concentrations of Cur-NLC (1, 2.5, and 5 mg/kg/day) + Cyp in groups IV-VI, respectively, showed a significant decrease in levels of ALT, AST (*p* < 0.01 and *p* < 0.001, respectively) in groups V and VI, and ALP (*p* < 0.001) in group VI as compared to Cyp (group III). The activity of these indices in Cur-NLC alone (Group II) is the same as in the control (Table 1). 

### 2.2. Effect on Albumin and Total Protein

A “significant” decrease in serum total protein and albumin level (*p* < 0.001) was observed in Cyp (group III) versus the control group (group I). Furthermore, co-treatment with varying concentrations of Cur-NLC (1, 2.5, and 5 mg/kg/day) + Cyp (50 mg/kg) in groups IV-VI, respectively, provided a significant increase in the values of total protein (*p* < 0.05; *p* < 0.01) in groups V and VI, as well as an increase in the levels of albumin (*p* < 0.01; *p* < 0.001) in groups V and VI as compared with Cyp alone (group III) (Table 1). The levels of these parameters in Cur-NLC alone (Group II) were the same as in the control.

### 2.3. Effects on LPO and GSH

**Figure 1:**
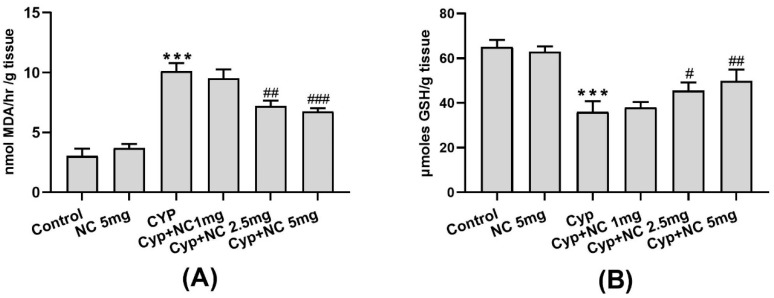
Cur-NLC treatment attenuated the level of LPO (**A**) and GSH (**B**). Values shown as mean ± SEM (n = 6). *** *p* < 0.001 vs. control, **^#^** *p* < 0.05, **^##^** *p* < 0.01, **^###^** *p* < 0.001 vs. Cyp group. Cyp = cypermethrin; NC = NLC.

Exposure to Cyp produced significant effects on the oxidative status of liver tissue, as shown by a significant rise in MDA level (*p* < 0.001) and a decrease in GSH (*p* < 0.001) (Figure 1A,B) compared to the control. However, supplementation of Cur-NLC along with Cyp significantly ameliorated the LPO (*p* < 0.01; *p* < 0.001) and GSH (*p* < 0.05; *p* < 0.01) level in groups V and VI as compared to Cyp-treated rats (group III). 

### 2.4. Effects on Antioxidative Enzymes (SOD and Catalase)

**Figure 2:**
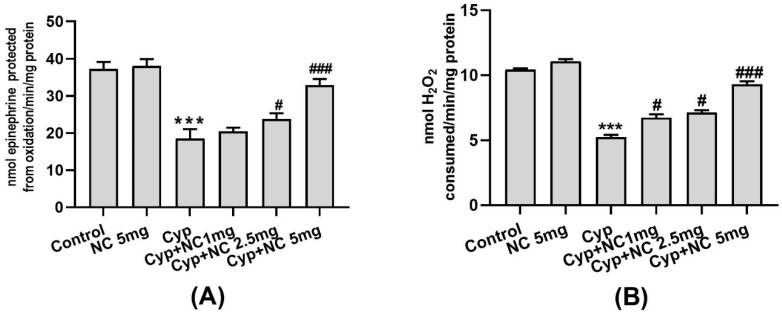
Cur-NLC treatment boost activities of SOD (**A**) and catalase (**B**). Data presented as mean ± SEM (n = 6). *** *p* < 0.001 vs. control, **^#^** *p* < 0.05, **^###^** *p* < 0.001 vs. Cyp group. Cyp = Cypermethrin; NC = NLC.

The levels of SOD and catalase in livers of Cyp-treated exposed rats (group III) resulted in a significant decrease in SOD and catalase activity (*p* < 0.001) (Figure 2A,B) in liver tissue as compared to the control (group I). Co-treatment of Cur-NLC with Cyp in rats significantly ameliorates the oxidative enzymes (SOD and catalase) activities (*p* < 0.05; *p* < 0.001) similar to the control in a dose-dependent manner.

### 2.5. Inflammatory Markers

**Figure 3:**
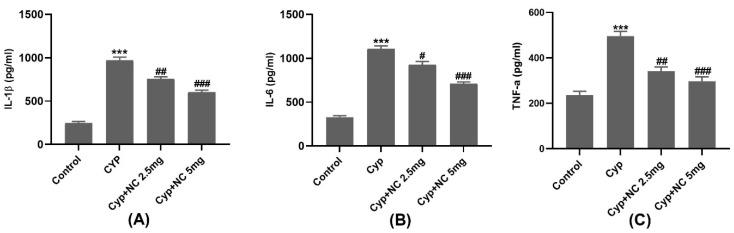
Cur-NLC treatment suppressed IL-1 β (**A**), IL-6 (**B**), and TNF-α (**C**) expression induced by Cyp. Value expressed as mean ± SEM (n = 6). *** *p* < 0.001 vs. control, **^#^** *p* < 0.05, **^##^** *p* < 0.01, **^###^** *p* < 0.001 vs. Cyp group. Cyp = cypermethrin; NC = NLC.

Cyp induces raised expression of interleukins (IL-1β, IL-6, and TNF-α) in Cyp alone (group III) (*p* < 0.001) (Figure 3A–C). Moreover, co-treatment of Cur-NLC significantly ameliorates the expression of cytokines (IL-1β, IL-6) in the Cyp + Cur-NLC (groups V and VI) groups (*p* < 0.05, *p* < 0.01, *p* < 0.001); however, the expression TNF-α is decreased in groups V and VI (*p* < 0.01, *p* < 0.001, respectively). All cytokines in Cur-NLC (group II) are the same as in the control. 

### 2.6. Assay on Caspases 3 and 9

**Figure 4:**
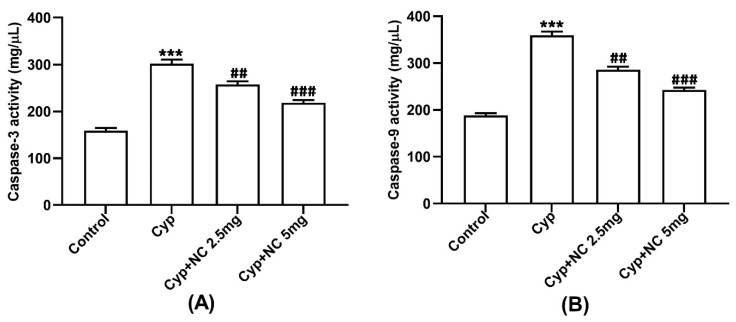
Effect of Cur-NLC treatment on apoptotic markers caspase-3 (**A**) and caspase-9 (**B**) up-regulated by Cyp. Values represented as mean ± SEM (n = 6). *** *p* < 0.001 vs. control, **^##^** *p* < 0.01, **^###^** *p* < 0.001 vs. Cyp group. Cyp = cypermethrin; NC = NLC.

Cyp induces raised expression of *caspases* 3 and 9 in the Cyp-only group (III) (*p* < 0.001) (Figure 4A,B). Moreover, co-treatment of Cur-NLC significantly ameliorates the expression of caspases 3 and 9 in the Cyp + Cur-NLC groups (V and VI) (*p* < 0.01, *p* < 0.001, respectively).

### 2.7. Immunohistochemical Studies

**Figure 5:**
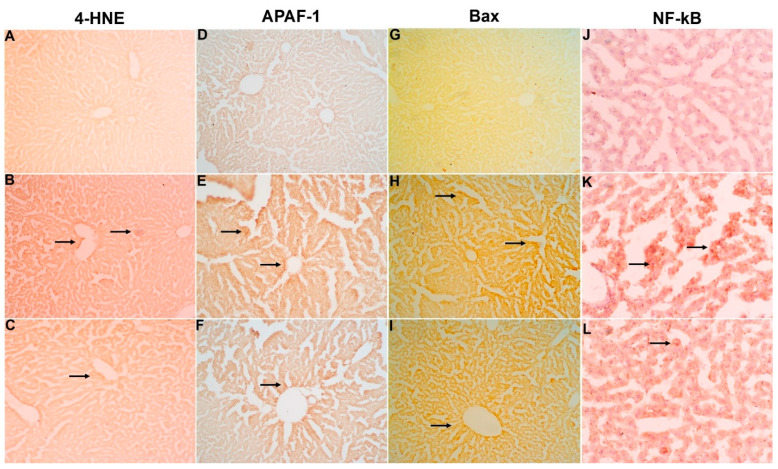
Micrograph representing the protective role of Cur-NLC in relation to LPO marker 4-HNE (**A**–**C**), apoptotic indices (APAF-1 and Bax, **D**–**I**), and inflammatory marker NF-κB (**J**–**L**) by immunostaining. Elevated expression was observed in the section of the Cyp-treated group (**B**,**E**,**H**,**K**) as compared to the control (**A**,**D**,**G**,**J**), while the Cyp+NC (5 mg)-treated group has shown a suppression in immunostaining of 4-HNE (**C**), APAF-1 (**F**), Bax (**I**), and NF-κB (**L**). Photomicrographs were obtained by light microscope at 40× magnification.

Upon Cyp treatment, a profound expression of 4-HNE (Figure 5B), Apaf-1 (Figure 5E), Bax (Figure 5H), and NF-κB (Figure 5K) were reported in liver tissues (group III). At the same time, co-treatment of Cur-NLC showed a marked amelioration of expression with varying doses. The tissue with the highest dose of NC (5 mg) (Figure 5C,F,I,L) (group VI) shows a nearly similar pattern to the control (group III) (Figure 5A,D,G,J). However, doses of 1 mg and 2.5 mg (groups VI and V, respectively) do not show any observable change when compared to the Cyp group.

### 2.8. Quantitative DNA Estimation

**Figure 6:**
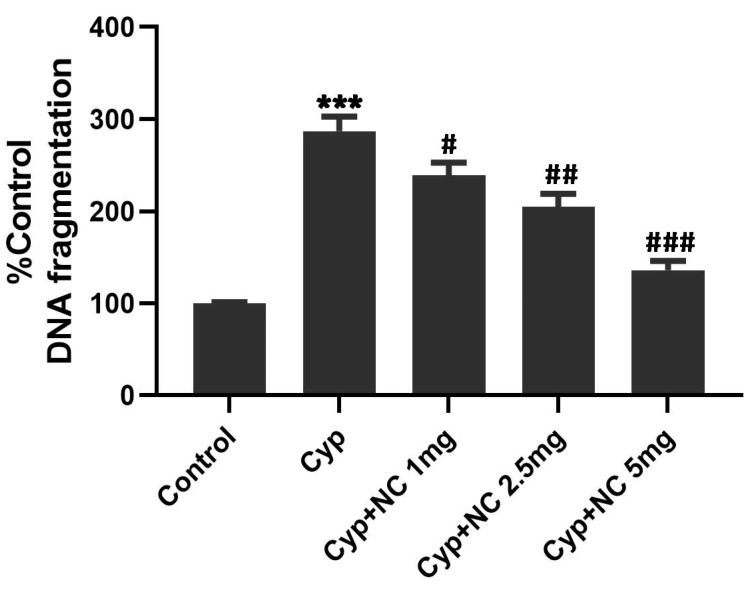
Effects of Cur-NLC on Cyp induced DNA fragmentation in the liver. DNA represented by nucleosomal fragments that show the degree of fragmentation induced by agents alone or in combination. Results are mean ± SEM with n = 5 rats per group. Values expressed as *** *p <* 0.001 vs. control, **^#^** *p <* 0.05, **^##^** *p <* 0.01, **^###^** *p <* 0.001 vs. Cyp-treated group. Cyp = cypermethrin; NC = NLC.

The inhibitory effect of Cur-NLC in pre-exposure with Cyp on genomic integrity is shown in Figure 6. In agreement with previous research [20], Cyp toxicity leads to a rise in DNA fragmentation of up to 287.45% (*p <* 0.001) compared with the control; however, pretreatment Cur-NLC with doses of 1, 2.5, and 5 mg restrict this fragmentation to 239.68, 205.19, and 136.47% (*p <* 0.05, *p <* 0.01, *p <* 0.001), respectively, as compared to the control.

### 2.9. DNA Fragmentation by Agarose Gel Electrophoresis

**Figure 7:**
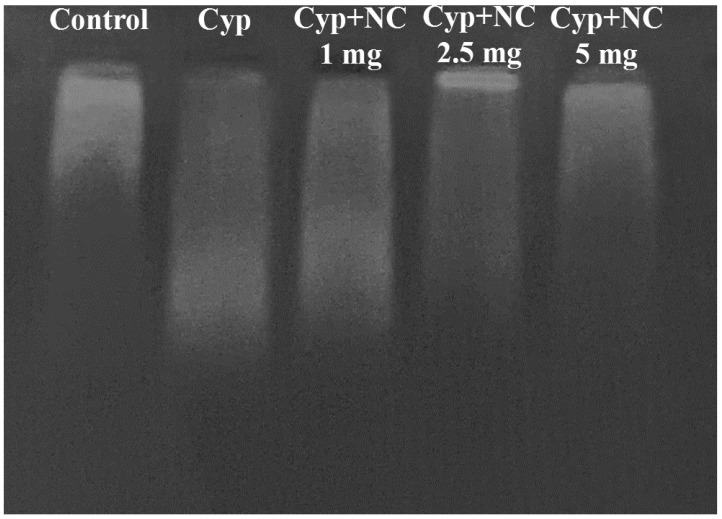
Cur-NLC protects genomic DNA fragmentation in the liver. Lanes are as follows: lane 1: control; lane 2: Cyp; lane 3 Cyp + Cur-NLC (1 mg); lane 4: Cyp + Cur-NLC (2.5 mg) lane 5: Cyp + Cur-NLC (5 mg). Cyp = cypermethrin; NC = NLC.

Based on qualitative changes in the integrity of DNA (Figure 7), in the control group (first lane), some DNA fragmentation was observed. Animals treated with Cyp alone in group III show massive fragmentation in terms of smear-like pattern (second lane), which represents extensive toxicity. The length of smear is reduced in groups IV to VI (third to fifth lane) and corresponds to the doses 1, 2.5, and 5 mg of Cur-NLC. At a dose of 5 mg, the pattern of smear is almost similar to the control, reflecting the ability of Cur-NLC to reverse the Cyp-induced DNA damage.

### 2.10. Histopathology

**Figure 8:**
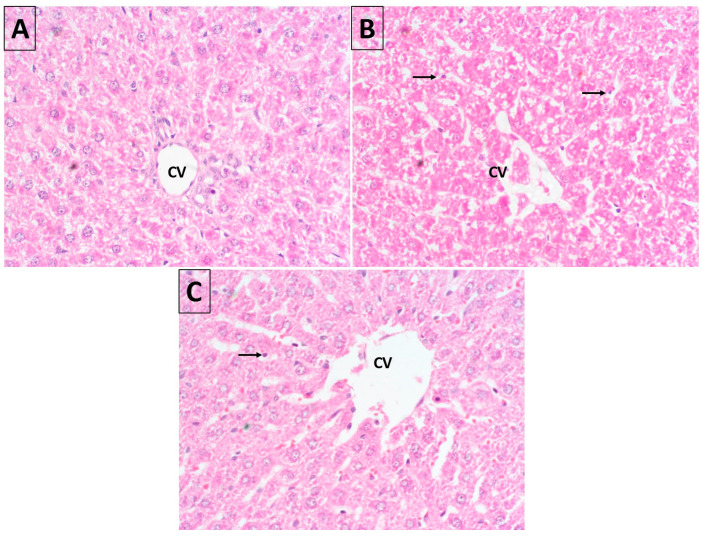
Morphological changes were observed by H&E staining induced by Cyp. Normal cell morphology and central hepatic vein were detected in the control group (**A**), while treatment with Cyp shows accumulation of inflammatory cells in the portal triads which had pyknotic nuclei (**B**). However, the co-treatment of Cur-NLC shows the reversal of histopathological changes with partial cell damage at a 5 mg Cur-NLC dose (**C**). (Data from 1 mg and 2.5 mg not shown.) Magnification, 40×.

The histopathological studies (Figure 8A) showed that normal architecture of hepatic cells, clear sinusoidal spaces, and a central vein were observed in the control groups. Cyp treatment causes drastic histopathological alterations at various stages of degeneration in the hepatocytes, including severe parenchymal architectural disruption, dilatation of sinusoids, and central vein congestion (Figure 8B). These findings are in accordance with the results [33]. Upon Cur-NLC co-treatment, the distorted structure of liver tissue again normalizes at a level close to the control group (Figure 8C).

## 3. Discussion

We determined the beneficial effect of Cur-NLC on Cyp-induced toxicity in rat liver. Reactive oxygen species produced by Cyp exposure can compromise cellular antioxidative defense order and concurrently injure the cellular macromolecules, including lipids, proteins, and DNA. The present work showed that Cyp administration instigates oxidative imbalance in rat liver as authenticated by antioxidative markers and might be treated with curcumin.

Previous works indicated that Cyp-induced toxicity leads to an increase in the intracellular levels of reactive oxygen species and finally oxidative stress [34,35]. The increased AST, ALT, and ALP, and decreased total protein and albumin activities are due to the oxidative damage by ROS. AST is usually found in several organs including the liver, heart, muscles, kidneys, and brain, and is released into the bloodstream when tissue is damaged. ALT is mainly present in the liver and is transmitted into the bloodstream due to liver injury. The increase in AST and ALT activities in serum in our study is in agreement with the previous findings of Yousef et al. (2010) [36].

A reduced level in total protein and albumin after Cyp toxicity was also earlier reported by Yousef et al. (2003), who propose that the decrease in total protein was mainly due to a reduction in albumin instead of globulin fraction [37]. Rivarola and Balengo deduced that the decrease in plasma protein, especially albumin, by pesticide treatment might be attributed to changes in protein metabolism in the liver [38]. The changing serum levels of albumin thus provide valuable evidence of acuteness, progression, and prognosis in liver disease [39].

LPO plays a primary function in the etiology of diseases. It is responsible for disturbing the permeability of membranes, leading to leakage of enzymes [40]. LPO is initially carried out by free radicals and leads to cellular damage by deactivating membrane enzymes, depolymerization of polysaccharides and breakdown of proteins. Tissue injuries in rats and rabbits due to the exposure of pyrethroids have already been reported [41,42]. Our results (Figure 1) clearly demonstrate that Cyp toxicity caused a marked rise in LPO with a reduction in antioxidant enzyme activity (SOD, CAT) in the liver.

These results are also authenticated by the decreased level of GSH in the liver reflecting its oxidative stress because glutathione is a tripeptide that possesses cysteine amino acid [43]. It appears that depletion of GSH levels in the liver is due to toxic Cyp initiated by the production of ROS followed by an oxidative imbalance that leads to cell death [44].

Furthermore, the reduced level of GSH in tissues after Cyp treatment might be due to three possible mechanisms: (i) in Cyp metabolism, GSH is the primary substrate; (ii) Cyp binds with the thiol group of GSH; (iii) GSH is converted to GSSG by Cyp-induced free radical-mediated oxidation [45].

Treatment with Cur-NLC with varying doses effectively reduced LPO in terms of MDA and increased GSH levels in the co-treated groups. Because of the lipophilic nature of curcumin, its conceivably depressed LPO is initiated by free radicals, thus diminishing cell damage [46]. Cur-NLC also increases the membrane permeability that might increase the uptake of GSH into the cell by increased activity of the GSH transport mechanism, which leads to increased antioxidant status inside the cell [47]. A previous study reported that entrapped liposome for curcumin delivery shows antioxidant activity [48].

Antioxidative enzymes are believed to be the first defensive mechanism against oxidative damage. SOD and catalase are antioxidative enzymes, which counter free radical production [49]. The activities of SOD and catalase are decreased due to the production of ROS leading to an increase in lipid peroxidation. There is a marked reduction in SOD and catalase activities in liver and brain tissues [50] with the depletion of GSH and GPx in erythrocytes after cutaneous exposure of Cyp [51]. SOD is a metalloenzyme that converts superoxide anions into water and molecular oxygen. Catalase is a heme-enzyme, converting hydrogen peroxide (toxic) to oxygen and water (non-toxic). Activities of catalase in Cyp-treated animals are reduced due to the excessive generation of hydrogen peroxide. Moreover, it has been reported that LPO might be an important aspect of the reduction in the catalase activity in Cyp toxicity [52]. Treatment with Cur-NLC with varying doses effectively ameliorates the SOD and catalase activities in the co-treated groups.

The exact mechanism of action that leads to an increase in inflammatory markers and apoptosis due to ROS remains poorly understood. We determined that inflammatory cytokines, such as tumor necrosis factor (TNF-α), interleukin (IL-1β, IL-6), and apoptotic factors, such as caspases 3 and 9, are induced by the generation of ROS in injured liver cells. All these are polypeptides that exert pleiotropic actions on cells and regulate expression of genes necessary for defensive mechanism reactions and immune response [53,54]. This study demonstrates that TNF-α, IL-1β, IL-6, and caspases 3 and 9 over-produced by Cyp induced hepatotoxicity. Thus, our finding is also supported by previous research demonstrating that curcumin ameliorates Cyp-induced liver dysfunction [55].

Another mechanism of Cyp-induced liver injury associated with inflammation [56] is evaluated by immunohistochemistry. Increased expression of inflammatory markers (NF-κB), oxidative stress markers (4-HNE), and apoptosis markers (Bax and Apaf-1) represents an oxidative imbalance, and these protein expressions are increased in terms of stress conditions. In our study, the increase in the expression from Cyp toxicity is clearly seen by immunohistochemistry; however, upon concurrent treatment with Cur-NLC in a dose-dependent manner, the expression of 4-HNE, NF-κB, Bax, and Apaf-1 was reversed. Moreover, our results are similar to those of an earlier study [57].

Apoptosis can also be evaluated by looking at the DNA fragmentation pattern [20]. However, DNA breakdown is a phenomenon that follows cell rupture in necrosis, as the chromatin after digestion with proteolytic enzymes and endonucleases forms a smear-like pattern [58]. In the control group (first lane), very low DNA laddering appears (Figure 7). The DNA smear appears to be concentrated close to the well, suggesting no DNA fragmentation. In the Cyp-treated group (group III) (second lane), the extensive DNA fragmentations appear as DNA laddering (smearing). The appearance of smears in the co-treated group decreased in a dose-dependent manner. At a high dose of Cur-NLC (5 mg), the length of smear is reduced (fifth lane) similar to the control [59]. Our results can also be authenticated by quantitative DNA fragmentation in terms of percentage apoptosis. A decrease in percentage apoptosis in the co-treated group from groups IV to VI shows the antiapoptotic effect of curcumin.

Cyp toxicity in rat liver is also evaluated by histopathological studies, which revealed that the liver showed a high degree of retrieval, and liver cells maintained their normal architecture, although in central veins some degree of swollenness still appeared upon Cur-NLC treatment. The marked histopathological changes due to Cyp toxicity were sinusoidal spaces that were not uniform, distorted cell morphology and dilation and congestion of the central vein (Figure 8). Our findings are also in agreement with a previous study by Manna et al., 2004 [33]. All the changes were ameliorated similarly to the control at the highest dose (5 mg) of Cur-NLC. These results also provide strong confirmation that Cur-NLC can be applied for the treatment of toxic hepatopathy.

In the present study, Cyp toxicity induces the generation of ROS, which leads to oxidative stress (LPO, GSH, 4-HNE). These oxidative changes are implicated in the development of apoptosis through the induction of APAF-1, DNA fragmentation, caspases 3 and 9 expression. Furthermore, the Cyp exposure leads to induction of inflammatory responses in the liver tissues, evident from the upregulated levels of IL-1β, IL-6, NF-kβ and TNF-ɑ. These potent pro-inflammatory cytokines act as master regulators of inflammation. Upon Cur-NLC treatment, all oxidative stress, inflammatory, and apoptotic markers were reversed close to the control at higher doses. Thus, our results are also validated by various in vitro studies [60,61].

In conclusion, the observations from this study strongly support the hepatoprotective effect of Cur-NLC against Cyp-induced hepatotoxicity. Cyp induced toxicity by triggering oxidative stress, inflammation, and apoptosis. These effects may be due to the improved distribution in the liver as a result of enhanced antioxidant, anti-inflammatory, and antiapoptotic activities of Cur-NLC. Finally, Cur-NLC could help protect people and animals at risk after Cyp exposure.

## 4. Materials and Methods

### 4.1. Chemicals

In this study, commercially available analytical chemicals of the highest grade were procured from Sigma Chemicals, USA. All ELISA kits for calorimetric estimation of IL-6, IL-β, TNF-α, Bax, caspases-3,9, NF-κB, and all antibodies for immunohistochemistry were purchased from Abcam, Cambridge (UK). Kits for estimation of ALT, AST, ALP, total protein, and albumin in serum were purchased from Randox, County Antrim (UK).

### 4.2. Animals

Wistar male rats (200–230 g) were taken from the animal house at Jazan University. This study was approved by the Institutional Research Review and Ethics Committee (IRREC, Letter Number: 308/203/1443), College of Pharmacy, Jazan University on 7 November 2021. Animals were kept in standard feed and laboratory conditions during the study.

### 4.3. Dose Selection

Cyp was freshly prepared by being dissolved in normal saline, and a dose of 50 mg/kg/day was supplied orally for 15 days [62,63]. Cur-NLC was administered orally for 10 days in three different doses (1, 2.5 and 5 mg/kg/day), and starting after 5 days, Cur-NLC was administered for 10 days with a time gap of one hour being maintained between both doses. TEM image and results of Cur-NLC was shown in Figures S1 and S2 respectively (Supplementary file).

### 4.4. Experimental Design and Sample Preparation

Rats were randomly divided into six groups, with eight animals in each one.

Group 1—untreated control group (control), given normal saline for 15 days.

Group 2—given orally, Cur-NLC at a dose of 5 mg/kg/day for 10 days.

Group 3—given orally, Cyp at a dose of 50 mg/kg/day for 15 days.

Group 4—received Cyp, 50 mg/kg/day for 15 days and co-treated with Cur-NLC (1 mg) for 10 days.

Group 5—received Cyp, 50 mg/kg/day for 15 days and co-treated with Cur-NLC (2.5 mg) for 10 days.

Group 6—received Cyp at a dose of 50 mg/kg/day for 15 days and co-treated with Cur-NLC (5 mg) for 10 days.

Toxicity symptoms were observed throughout the treatment regime. Before execution of the study, blood samples were collected from all group animals by ocular puncture. Each blood sample was centrifuged at 3000 rpm for 10 min to obtain serum for analyzing serum markers. After that, animals were euthanized by CO_2_ inhalation followed by cervical dislocation and the liver was isolated for 5–10% homogenate and post-mitochondrial supernatant (PMS) preparation in 10 mM phosphate buffer (pH 7.4). Biochemical assay was conducted in homogenate/PMS by using double-beam UV-spectrophotometer (UV-1800, Shimadzu, Kyoto, Japan). Inflammatory markers and caspase-3,9 was estimated by ELISA assay kit (Abcam) using a 96-well plate reader (ELx 800TM BioTek, Winooski, VT, USA). Animal liver tissue was preserved in 10% formalin for the histopathological study and immunohistochemistry. Separate liver tissue was collected for DNA isolation and fragmentation study by agarose gel electrophoresis.
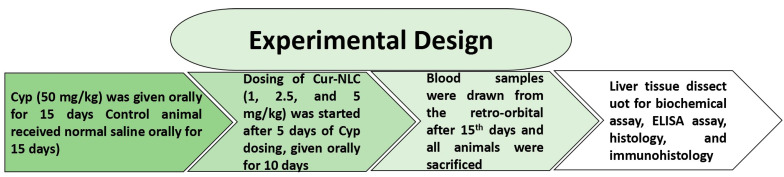


### 4.5. Serum Biomarkers Assay

The activity of ALT, AST, ALP, total protein, and albumin were determined by using a spectrophotometer, according to the protocol provided by Randox Pvt Ltd. (Crumlin, UK).

### 4.6. Estimation of LPO and GSH and Antioxidant Enzyme Activity

Estimations of LPO in terms of malondialdehyde (MDA) and GSH were carried out in liver tissue by preparing 10% homogenate [64]. The anti-oxidative enzyme (SOD and catalase) activity status was measured in the post-mitochondrial supernatant (PMS) of liver tissue. The protein concentration was originally measured by Lowry et al. (1951) [65].

### 4.7. Inflammatory Markers

Interleukin cytokines (IL-1beta, IL-6, and TNF-α) activities were estimated by protocol provided by ELISA Kit from Abcam, Cambridge (UK). The absorbance was taken at 405 nm by a 96-well plate reader (ELx 800TM BioTek, USA).

### 4.8. Apoptotic Markers Assay Caspases 3,9

Caspases were calorimetrically estimated by ELISA kits (Abcam, UK) according to the manufacturer’s protocol. Reading was performed at 450 nm by microplate reader (ELx 800TM Bio-Tek, USA).

### 4.9. Immunohistochemistry of 4-HNE, NF-κB, Bax, and Apaf-1

Immunohistochemistry was performed to detect the expression of 4-HNE, NF-κB, Bax, and Apaf-1 proteins. Liver sections were obtained after cryo-sectioning (10–20 µm thick). Tissue sections were washed with PBS and incubated for 1 h each with blocking solution to reduce endogenous peroxidase activity and block non-specific antibody binding. The sections were treated with primary and secondary antibodies. Slides were then exposed to DAB to develop color. The sections were covered with coverslip after dehydration and mounted with mounting media. Photomicrographs were taken by light microscope at 40× magnification [66].

### 4.10. Quantitative DNA Assay and DNA Fragmentation by Agarose Gel Electrophoresis

Quantitative DNA assay is used to determine the percentage DNA fragmentation [20], which can be further evaluated by agarose gel electrophoresis using 2% agarose. Liver samples stored at −80 °C were homogenized in pre-chilled 3 mL lysis buffer using a glass homogenizer. After centrifuging the homogenates at 27,000× *g* (4–8 °C), the pellets and supernatant were treated with 4 mL 0.5 N and 1 mL 5.5 N perchloric acid, respectively. Capped samples were then boiled for 20 min followed by sonication and 2nd centrifugation at 10,000× *g* for 10 min and aliquot 1 mL of the supernatants. Then, 4 mL of freshly prepared Burton’s Reagent (diphenylamine) was added to tubes and capped with Para film and kept in the dark for 16–24 h at room temperature. Absorbances were recorded at 600 nm using a UV/VIS Beckman DU-640 spectrophotometer and DNA fragmentation was calculated according to a previous method. A Qiagen DNA isolation kit (Qiagen Science, Maryland USA) was used to isolate genomic DNA. The manufacturer’s method was used in the process isolation for all treated tissues. Each DNA sample was loaded onto 2% agarose gel containing 0.1% ethidium bromide, and a laddering pattern was determined by running the gel at 60 V using a Large Submarine (Hoeffer Instruments, San Francisco, CA, USA). A photograph of the gels was captured on a UV transilluminator.

### 4.11. Histopathological Study

Liver tissue was dissected from each group and fixed in 10% formalin solution [67]. The formalin fixed tissue was dehydrated by soaking up alcohol and xylol. The fixed tissue was further processed for making paraffin block to obtain 5 µm-thick tissue sections by microtome (LEICA, RM2125RTS). After sectioning, we dewaxed the sections and processed them with hematoxylin and eosin staining. The sections were cleared in xylene and mounted in DPX mounting medium. The histological images were acquired using light microscopy (Olympus CX21, Tokyo, Japan) at 40× magnification.

### 4.12. Statistics

Data are represented as mean ± standard error, and analysis of variance followed by T-tests was used to measure the significance. The data were analyzed by using GraphPad Prism software (Version 8.0.2).

## Figures and Tables

**Table 1:** Biochemical effects of CUR-NLC in serum of Cyp-induced hepatotoxicity in rats.

Liver Markers	Control	CUR-NLC 5 mg	Cyp 50 mg	Cyp + CUR-NLC		
				1 mg	2.5 mg	5 mg
ALT (IU/L)	45 ± 6	43 ± 7	189 ± 12 ***	176 ± 10 ^#^	159 ± 10 ^##^	106 ± 8 ^###^
AST (IU/L)	36 ± 3	34 ± 4	89 ± 7 ***	69 ± 6 ^##^	56 ± 4 ^##^	50 ± 5 ^###^
ALP (IU/L)	89 ± 10	91 ± 11	172 ± 15 ***	153 ± 12 ^##^	147 ± 11 ^###^	123 ± 12 ^###^
Total protein (g/dL)	8 ± 1	8 ± 1	4 ± 1 ***	5 ± 1	7 ± 1 ^#^	7 ± 1 ^##^
Albumin	4 ± 1	4 ± 1	2 ± 2 ***	3 ± 1	3 ± 1 ^##^	4 ± 1 ^###^

Liver ALT, AST, ALP, total protein, and albumin significantly change following Cyp and Cur-NLC treatment. Values are expressed as mean ± S.E.M of n = 6 animals. *** *p* < 0.001 Cyp vs. control. ^#^ *p* < 0.05, ^##^ *p* < 0.01, ^###^ *p* < 0.001 Cyp + Cur-NLC vs. Cyp.

## Data Availability

The data available within the articles.

## Supplementary Materials

The following supporting information can be downloaded at: https://www.mdpi.com/article/10.3390/molecules28020881/s1, Figure S1. Micrograph of Cur-NLC obtained from transmission electron microscopy; Figure S2. Size (60.58 ± 4.16 d.nm) and zeta potential (−48.57 ± 2.61 mV (n = 3) of Cur-NLC (n = 3); Cur-NLC particle size distribution, average particle size distribution of one of three (n = 3) optimized formulations. The size of the particle was 60.58 ± 4.16 nm with narrow size distribution (<0.5). References [68,69] are cited in the supplementary materials.
