# Supplementary material for: Hepatoprotective Effect of Curcumin Nano-Lipid Carrier against Cypermethrin Toxicity by Countering the Oxidative, Inflammatory, and Apoptotic Changes in Wistar Rats
Source: Molecules. 2023 Jan 16;28(2):881. doi: 10.3390/molecules28020881 (PMC9864069; doi:10.3390/molecules28020881)
Supplement: Supplementary file 1 [file molecules-28-00881-s001.zip › molecules-2085278-supplementary.pdf]

Nano formulation of curcumin

Cur-NLC (curcumin nano-lipid carrier) was prepared by hot homogenization method [68]. The solid lipid (stearic acid) (500 mg) was heated in a beaker and sesame oil (200 µl) was mixed. Accurately weighed amount of curcumin (50 mg) was added and mixed this mixture. Another beaker containing water (25 ml) mixed with tween-80 (25 µl) and sodium dodecyl sulphate (50 mg) was heated up to 70°C. It was followed by homogenization of both the phases for 15 min at a speed of 5000 rpm. The image of Cur-NLC were taken by T*ransmission Electron Microscope Scanning (TEM – SCAN)* (Figure S1). Cur-NLC was characterized by the determination of particle size, polydispersity index (PDI), zeta potential.

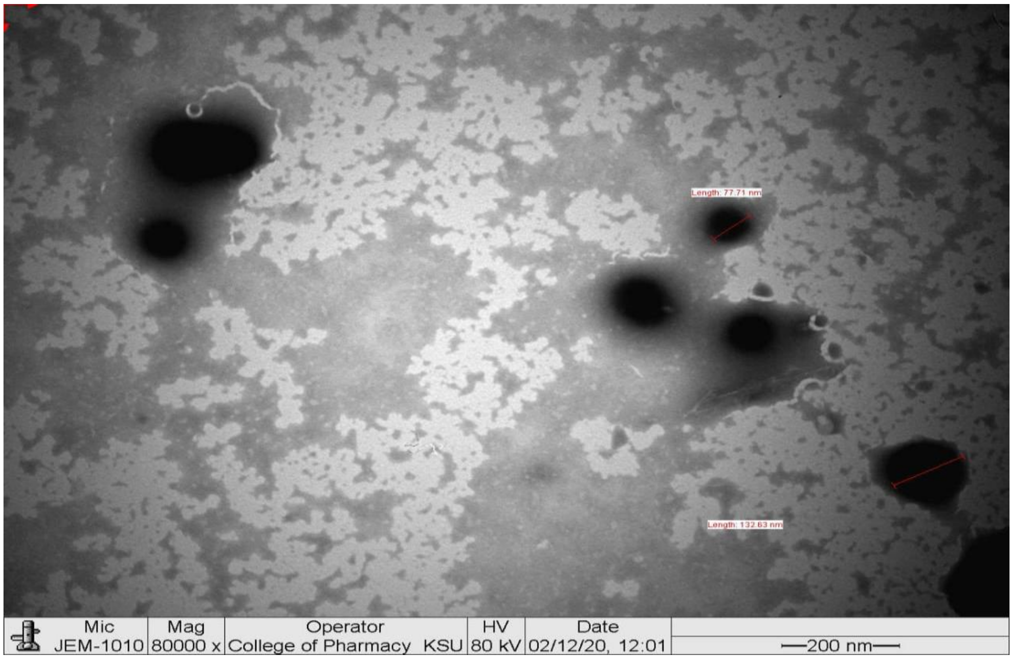

Figure S1. Micrograph of Cur-NLC obtained from transmission electron microscopy.

Particle size, polydispersity index and zeta potential

Cur-NLC was successfully prepared and characterized based on particle size, zeta potential and polydispersity index (PDI). The mean size of Cur-NLC was found to be 60.58 ± 4.16 d.nm (n = 3) (Figure S2). The PDI was found to be 0.252 ± 0.019 (n = 3).

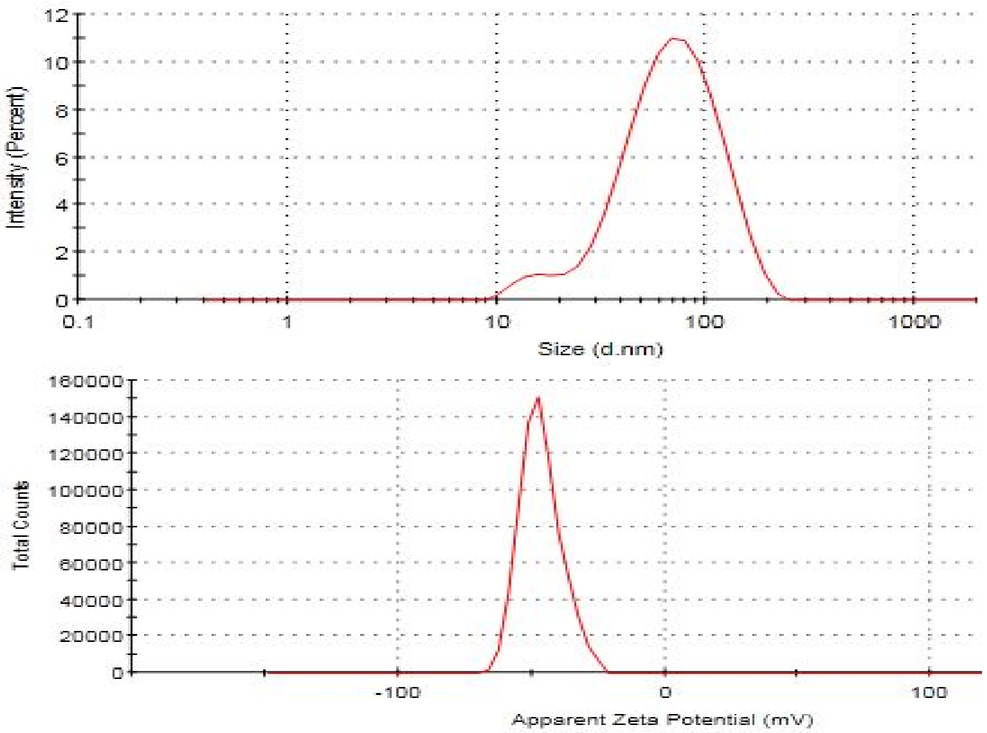

Figure S2. Size (60.58 ± 4.16 d.nm) and zeta potential (– 48.57 ± 2.61 mV (n = 3) of Cur-NLC (n = 3); Cur-NLC particle size distribution, average particle size distribution of one of three (n = 3) optimized formulations. The size of the particle was 60.58 ± 4.16 nm with narrow size distribution (< 0.5).

The optimum clinical outcomes can be achieved with a constant and narrow size distribution of nano-carrier formulations. The narrow size distribution is achieved by the smaller value of PDI [69]. Thus, Cur-NLC is considered as nano-formulations with narrow size distribution.

The surface charge of NLC was characterized by determining the zeta potential. The zeta potential of Cur-NLC was found to be – 48.57 ± 2.61 mV (n = 3). The surface charge is considered to be a key factor for the stability of colloidal dispersions. The zeta potential value of greater than +/- 20 mV is required for electrostatic stabilization [68]. Cur-NLC exhibited larger zeta potential value indicating its stability for longer duration of time.

References

68. Makeen,, H.A.; Mohan, S.; Al-Kasim, M.A.; Sultan, M.H.; Albarraq, A.A.; Ahmed, R.A.; Alhazmi, H.A.; Alam, M.I. Preparation, Characterization, and Anti-Cancer Activity of Nanostructured Lipid Carriers Containing Imatinib. *Pharmaceutics* **2021**, *13*, 1086.
69. Makeen, H.A.; Mohan, S.; Al-Kasim, M.A.; Ibraheem M.A.; Ahmed, R.A.; Syed, N.K.; Sultan, M.H., Al-Bratty, M.; Alhazmi, H.A.; Safhi, M.M.; Ali, R.; Alam, M.I. Gefitinib loaded nanostructured lipid carriers: Characterization, evaluation and anti-human colon cancer activity in vitro. *Drug Delivery* **2020**, *271*, 622-631.
